# Osseointegration of Alkali-Modified NANOZR Implants: An In Vivo Study

**DOI:** 10.3390/ijms20040842

**Published:** 2019-02-15

**Authors:** Satoshi Komasa, Mariko Nishizaki, Honghao Zhang, Seiji Takao, Derong Yin, Chisato Terada, Yasuyuki Kobayashi, Tetsuji Kusumoto, Shigeki Yoshimine, Hiroshi Nishizaki, Joji Okazaki, Luyuan Chen

**Affiliations:** 1 Department of Removable Prosthodontics and Occlusion, Osaka Dental University, 8-1, Kuzuhahanazono-cho, Hirakata-shi, Osaka 5731121, Japan; komasa-s@cc.osaka-dent.ac.jp (S.K.); nishizaki-m@cc.osaka-dent.ac.jp (M.N.); takao-s@cc.osaka-dent.ac.jp (S.T.); yin-d@cc.osaka-dent.ac.jp (D.Y.); terada-c@cc.osaka-dent.ac.jp (C.T.); yosimine@cc.osaka-dent.ac.jp (S.Y.); joji@cc.osaka-dent.ac.jp (J.O.); chen-luyuan900115@foxmail.com (L.C.); 2 Osaka Research Institute of Industrial Science and Technology Morinomiya Center, 1-6-50, Morinomiya, Joto-ku, Osaka-shi 5368553, Japan; kobaya@omtri.or.jp; 3 Osaka Dental University Japan Faculty of Health Sciences, 1-4-4, Makino-honmachi, Hirakata-shi, Osaka 5731144, Japan; kusumoto@cc.osaka-dent.ac.jp (T.K.); nisizaki@cc.osaka-dent.ac.jp (H.N.)

**Keywords:** NANOZR, alkali treatment, in vivo study

## Abstract

Ingredients and surface modification methods are being continually developed to improve osseointegration of dental implants and reduce healing times. In this study, we demonstrate in vitro that, by applying concentrated alkali treatment to NANOZR with strong bending strength and fracture toughness, a significant improvement in the bone differentiation of rat bone marrow cells can be achieved. We investigated the influence of materials modified with this treatment in vivo, on implanted surrounding tissues using polychrome sequential fluorescent labeling and micro-computer tomography scanning. NANOZR implant screws in the alkali-treated group and the untreated group were evaluated after implantation in the femur of Sprague–Dawley male rats, indicating that the amount of new bone in the alkali-modified NANOZR was higher than that of unmodified NANOZR. Alkali-modified NANOZR implants proved to be useful for the creation of new implant materials.

## 1. Introduction

Dental implants are prostheses used in tooth defect cases [1,2,3,4]. Commercially, pure titanium is a highly compatible material for dental implants [5,6] and has long been used as an implant material with high patient satisfaction [7]. Success at the time of implant implantation is directly attributable to the principle of osseointegration, which is the process of interaction between the implant and the implant surrounding tissue [8]. Surface properties of biomaterials such as pure titanium have a great influence on osseointegration. Various surface modification methods have been developed to restore the tissue surrounding implants. Increasing the surface roughness of the material surface increases the rate of bone formation [9] and shortens the healing time [10].

A previous study showed that nanostructural modification on the material surface improves the initial adhesion of bone marrow cells [11]. Nanostructure control of the material surface can shorten the osseointegration period of the implant material. It has been shown that the surface roughness affects the initial cell response such as α5 integrin interactions [12]. Surface properties and surface structure play an important role in improving protein adsorption, which in turn is due to the behavior of bone marrow cells.

Recently, it was shown that titania nanosheet (TNS) structures fabricated on titanium surfaces by treatment in 10 M NaOH aqueous solution at 30 °C [13]. Alkali treatment modified titanium surface smoothed a rough surface at the nanometer level [14]. TNS produced by chemical processing has been previously reported to promote the bone differentiation of rat bone marrow cells [14,15,16]. In addition, we reported that TNS-modified titanium surface is due to initial adhesion and differentiation induction of rat periodontal ligament cells and improvement in the initial adhesion of vascular endothelial cells. This material shows that this structure is useful for periodontal tissue regeneration and angiogenesis after wound healing [17,18,19]. It has been hypothesized that alterations in the TNS structure affect the behavior of multiple cell types. Therefore, TNS–modified titanium surface promote the improvement of protein adsorption such as amelogenin.

In terms of esthetic appearance, the issue with titanium implants is that the titanium color permeates and the color tone of the gingiva becomes dark in portions where bones and gums are thin [20,21]. Additionally, due to retraction of the surrounding tissue around the implant, the head of the implant may become visible over time. Zirconia implants provide metal-free treatment options for patients. NANOZR is a nanocomposite in which nanometer-sized alumina particles and ceria-stabilized zirconia particles are dispersed in ceria-stabilized zirconia crystals and alumina crystals, respectively. At present, it exhibits higher bending strength and fracture toughness value than the yttria-type zirconia widely used in dentistry by Nawa et al. [22,23,24]. In addition, when compared with 3Y-TZP, NANOZR has a resistance to low-temperature degradation, which is a disadvantage of zirconia [24,25]. The fatigue strength using the cycle test of NANOZR shows double the numerical value of 3Y-TZP [26].

According to the report of Li et al., the apatite layer that formed on the surface of titanium and titanium alloy by alkali treatment was involved in osseointegration [27]. Various reports have shown that acid treatment brings about changes in the layer of oxide and surface structure of titanium and zirconia surfaces [28,29,30]. As in this report, sand blasting treatment is known to change the surface roughness of titanium or zirconia surfaces. In our previous research reports, concentrated alkali treatment on titanium and titanium alloy was useful for hard tissue differentiation induction [14,15,16,31]. These results indicate that the surface structure change is useful as it possibly affects protein adsorption and cell behavior. We clarified in vitro that osseointegration can be accelerated by adapting the alkali treatment that we have so far used on the surface of NANOZR. Alkali-modified NANOZR is useful for improving initial adhesion and hard tissue differentiation induction of rat bone marrow cells compared to unmodified NANOZR surfaces [32,33]. These results suggest that alkali-modified NANOZR is useful as a novel implant material in vitro as well as the use of alkali-modified titanium, which has been previously reported [33]. These studies have potential for NANOZR implants as a new treatment option for metal allergy patients requiring implant treatment. However, clinical applications require analysis in vivo.

This study aimed to analyze how the alkali-modified NANOZR implants affect the hard tissue formation of implanted surrounding tissues in vivo compared to unmodified NANOZR implants.

## 2. Results

### 2.1. Surface Characterization

**Figure 1:**
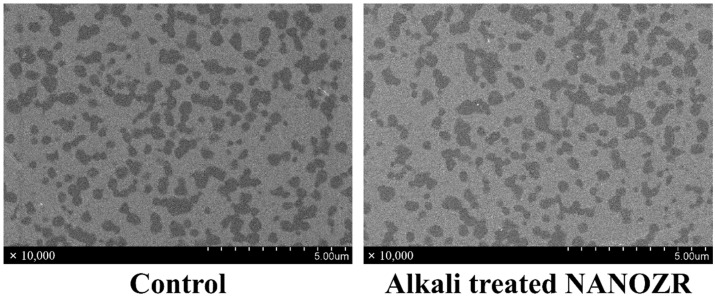
SEM micrographs of control and test groups.

In the scanning electron microscope (SEM) observation, there was no difference in the structure of the material surface between the alkali-modified NANOZR and the unmodified NANOZR (Figure 1).

**Figure 2:**
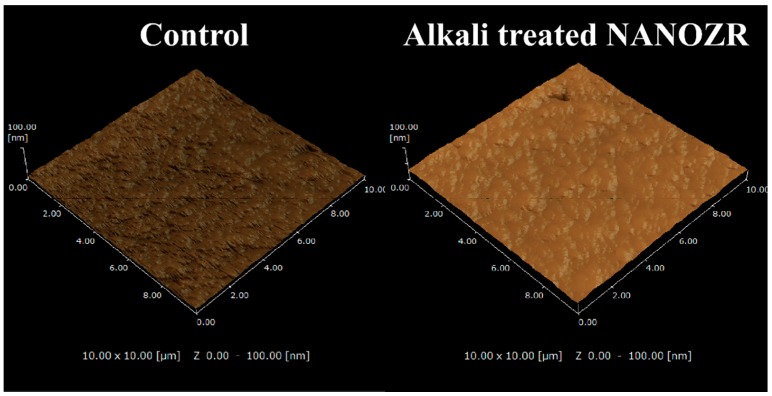
Scanning probe (SP) micrographs of control and test groups.

Scanning probe microscopy (SPM) analysis (Figure 2) showed that the Ra (2.3 nm) of the alkali-modified NANOZR surface was higher than the unmodified NANOZR (1.2 nm). (Ra = 1.2 nm).

**Figure 3:**
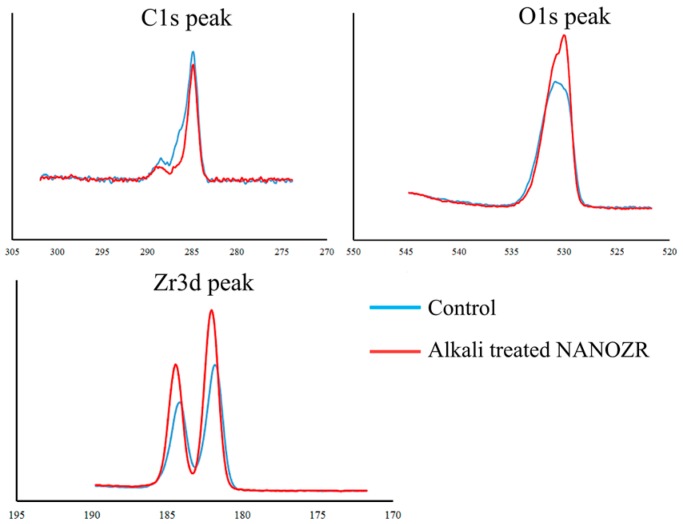
C1s, O1s, Zr3d XPS spectrum of the NANOZR surface (red line: control; blue line: alkali-treated NANOZR).

In the X-ray photoelectron spectroscopy (XPS) analysis, the intensity of the O1s peaks increased, while those of the Zr3d and C1s peaks decreased with NaOH treatment. The Na-peak was not assigned (Figure 3). Zeta potential of alkali-modified NANOZR implants were positive (5.3 ± 1.2), but those of unmodified NANOZR implants were negative (−18.4 ± 4.3).

### 2.2. Alkali Treatment-Induced Bone Differentiation on the NANOZR Surface In Vivo

**Figure 4:**
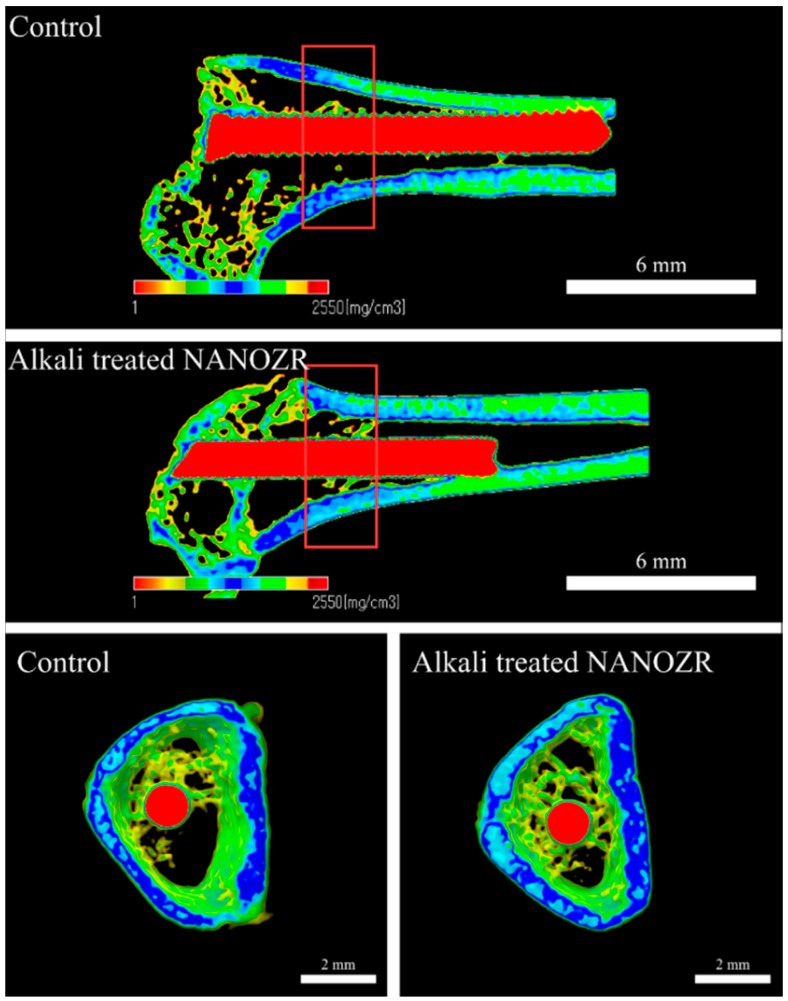
Micro-CT images (the implants are marked with red, cortical bone with blue, and cancellous bone with green).

Figure 4 shows a three-dimensional micro CT image reconstructed from the cross section of the sample eight weeks after implantation.

**Figure 5:**
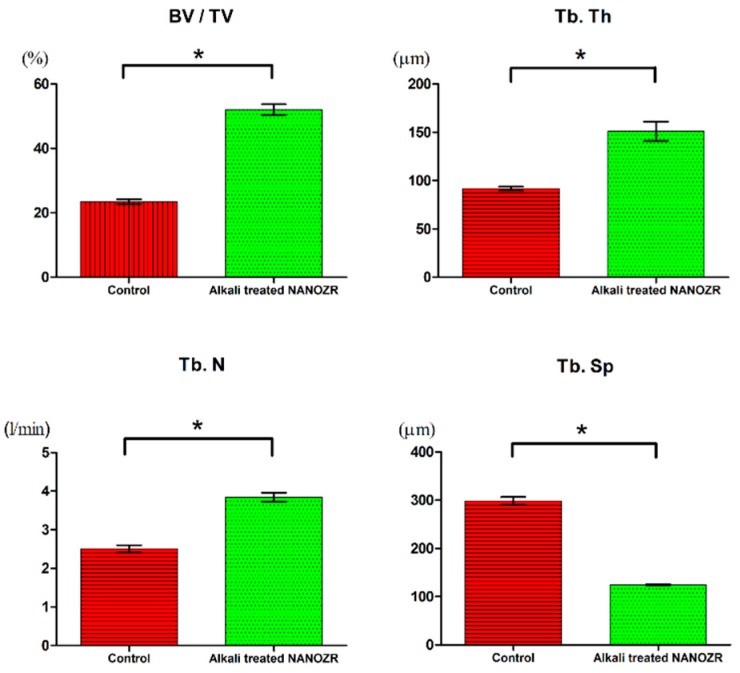
Quantitative evaluation of the trabecular bone within ROI (BV/TV, Tb.N, Tb.Th, and Tb. Sp). * *p* < 0.05.

Increased neonatal bone was found in the tissues surrounding the implants in both alkali-modified NANOZR surface and unmodified NANOZR surface groups. However, compared to the unmodified NANOZR group, the formation of high new bone was observed in the alkali-modified NANOZR group. Results of quantitative evaluation by image analysis are shown separately (Figure 5). BV/TV, Tb, N, and Tb values were significantly higher in the alkali-modified NANOZR group than in the unmodified NANOZR group (*p* < 0.05). Sp was significantly lower in the alkali-modified NANOZR group than in the unmodified NANOZR group.

**Figure 6:**
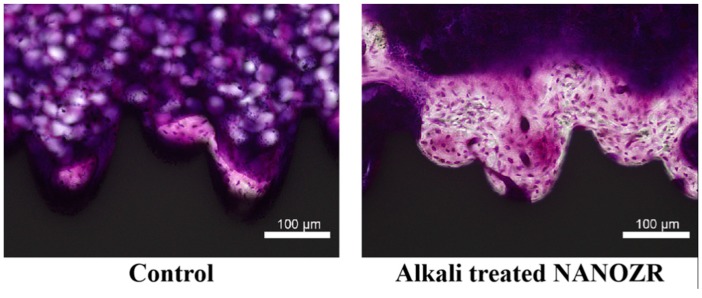
The longitudinally undecalcified histological sections with implants and peri-implant bones (purple: osteoid; white: new bone).

Undecalcified histological sections eight weeks after implantation are shown in Figure 6.

**Figure 7:**
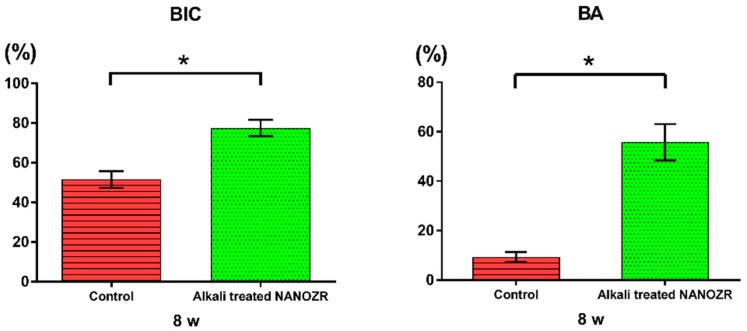
Quantitative histomorphometric analysis within the measured region (bone-implant contact (BIC) and bone area ratio (BA)). * *p* < 0.05.

The amount of new bone formed on the surface of alkali-modified NANOZR implants was higher than that of unmodified NANOZR implants. In the quantitative histomorphometric analysis of nondecalcified histological sections (Figure 7), the % BA value was significantly higher in the surrounding tissue of the implanted alkali-modified NANOZR implant compared with the unmodified NANOZR implant group (*p* < 0.05).

**Figure 8:**
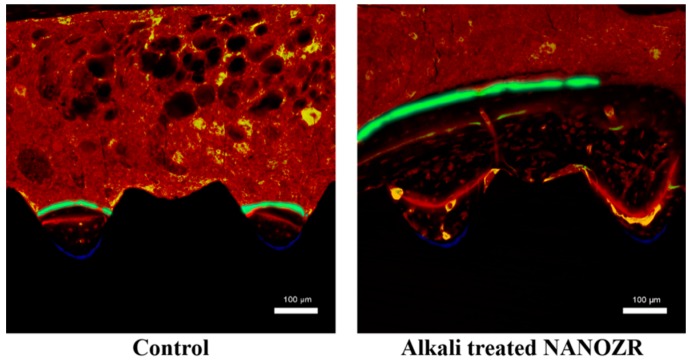
The histological sections were also observed using confocal laser scanning microscopy for dynamic histomorphometry according to fluorescence labeling (blue: 1 week of new bone, yellow: 4 week of new bone, green: 8 week of new bone, red: osteoid).

Histological sections were also observed using confocal laser scanning microscopy for dynamic histomorphometry as shown by fluorescence labeling (Figure 8).

**Figure 9:**
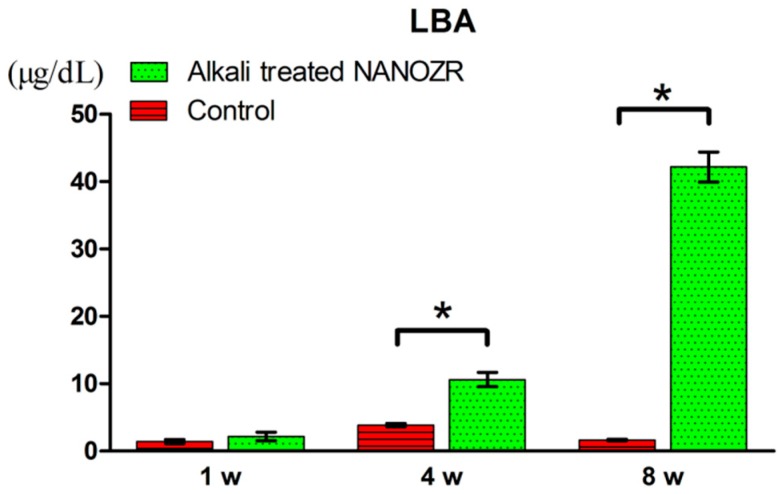
The percentage of labeled bone area (%LBA). * *p* < 0.05.

Three colored lines of blue (oxytetracycline hydrochloride, one week), red (Alizarin red S, four weeks), and green (calcein, eight weeks) were found in the implanted surrounding tissues. There was no difference in the amount of new bone around alkali-modified NANOZR implant and unmodified NANOZR implant one week after implantation. However, the new bone mass of surrounding tissues implanted at 4 and 8 weeks after implantation was significantly higher than that of unmodified NANOZR implants in alkali-modified NANOZR implants. According to the quantitative analysis, the numerical value of LBA was significantly higher in alkali-modified NANOZR implants than in unmodified NANOZR implants (Figure 9).

## 3. Discussion

In this study, we analyzed the influence of NANOZR implants subjected to surface modification by alkaline treatment on the implant surrounding tissues in vivo. Compared with the untreated polished NANOZR surface, it was revealed that the amount of new bone formation in the implanted surrounding tissues was larger in the alkali-modified NANOZR implant implantation group. This result suggests that alkali treatment to NANOZR is useful as an implant material, as well as the alkali-treated pure titanium that we have reported previously.

Zirconia is a novel ceramic material with excellent mechanical properties, high fracture toughness, and flexural strength [34]. Excellent stability in vivo, high strength, and elasticity are reasons for zirconia to be applied as a dental material [22,23,35]. Zirconia is useful as an implant material for metal allergy patients, as zirconia provides biocompatibility without causing inflammation in the tissues surrounding the implant [36,37]. Since NANOZR has higher fracture toughness than yttria-based zirconia, it is applied to denture base frames and implant materials [22,23]. We have demonstrated in vitro that ALP activity, OCN production, calcium deposition, and bone formation-related gene expression of rat bone marrow cells are improved by subjecting NANOZR material surfaces to alkaline treatment [32,33]. We have also reported that the hard-tissue-forming ability of this material is almost equivalent to that of alkali-treated pure titanium metal material [32,33]. In order to clinically apply this material, we evaluated the hard tissue formation of tissues surrounding implants of alkali-modified NANOZR and unmodified NANOZR in vivo.

In our previous report, surface roughness changed with alkali treatment application to NANOZR material surfaces [32]. Various reports have already reported that the surface roughness of the zirconia material surface was responsible for improving the initial backing of bone marrow cells [34,35]. In this experiment, the surface roughness of alkali-modified NANOZR implants was higher than that of unmodified NANOZR implants (2.3 vs. 1.2 nm). It is clear that the difference in the surface roughness of the material surface is involved in the initial adhesion of various cells immediately after implantation [36,37]. Our results show that the change in the surface texture of zirconia is an important factor inducing the early establishment of osseointegration [32]. An SEM image showing Al_2_O_3_ particles encapsulating nano level ZrO^2^ particles was found on the surface of NANZOR material. Nano-sized particles have been shown to be involved in the initial adhesion of adhesive proteins, initial adhesion of bone marrow cells, and enhancement of differentiation-inducing capacity [38]. Compared with ordinary yttria-type zirconia, NANOZR seems to have bioactivity because it contains nano-level ZrO_2_ particles and Al_2_O_3_ particles. On the alkali-modified NANOZR implant surface, XPS analysis showed an increase in the O1s peak and a decrease in the Zr3d peak. In experiments using pure titanium metal and zirconia in general, it is reported that an increase in the oxide layer is involved in hard tissue formation. It is also clear that the peak of Zr3d observed on the material surface is a hydroxyl group formed by the alkali treatment. It has been reported that hydrophilic materials induce the initial adhesion of bone marrow cells, which is consistent with the results of this experiment [39]. Our past studies revealed that Na+ ions are encapsulated in the hydrogel layer formed on the alkali-treated pure titanium metal surface [13]. However, alkali-modified NANOZR material surface formed a hydrogel layer lacking Na+ ions [32]. In the XPS analysis, the decrease of the C1 peak was also observed on the alkali-modified NANOZR surface. It is already clear that C1s is a peak showing deposition of contamination on the material surface and that the decrease in the C1s peak is useful for improving the initial adhesion of bone marrow cells and the induction of hard tissue differentiation. In our study, the decrease in C1s on the alkali-modified NANOZR surface and the increase in O1s peak intensity were associated with an increase in bone differentiation around the implant material [32]. In the current study, the zeta potential of unmodified NANOZR was negative, but that of alkali-modified NANOZR was positive. Miyake et al. showed that bone serum albumin has a negative potential and that more BSA may be adsorbed on the alkali-modified NANOZR surface, as two ions of different labels are adsorbed to each other [40]. Our recent work has led to the conclusion that alkali-modified NANOZR increases the adhesion of bovine serum albumin and may promote bone differentiation around the implant tissue after transplantation [32]. The in vivo evaluation in this study used rat femur and provides an allegory to clinical situations as the treatment is in direct contact with the trabecular bone. Although healing at eight weeks after implantation is considered to be the final stage of wound healing in the rat model [41,42], the contact rate of implanted material and bone is significantly lower in the implant. It is thought that it is the key to whether the material surface reacts early with cells. Our in vitro evaluation shows that the initial adhesion of bone marrow cells and the ability to induce hard tissue differentiation on the alkali-modified NANOZR surface are higher as compared with the unmodified NANOZR surface [32]. Correlating with the results of this in vitro evaluation, it is clear in various in vivo analysis results that NANOZR implants treated with alkali have a higher hard tissue formation in the surrounding tissues. In unmodified NANOZR implants, hard tissue formation is minimal in the surrounding tissue of the implantation, and it is clear that surface control is essential. Rita et al. reported that bone implant contact, as measured by histomorphometry, was slightly better on titanium than on zirconia surfaces in vivo [43]. Although no significant difference was observed in the amount of hard tissue formation between the alkali-modified NANOZR and the alkali-treated titanium, the titanium method showed a significantly high value in our recent study [32]. In the current study, only a small amount of hard tissue formation was observed in the alkali-modified NANOZR after one week of embedding. Our past reports clearly show that high hard tissue formation is observed around pure titanium metal, which was subjected to alkali treatment after one week of implantation, and it seems that the start of bone formation is slower in NANOZR. However, multiple reports [44,45,46] stating that the biocompatibility of the pure titanium metal surface and the zirconia surface are similar, and our observation of increased hard tissue formation after four weeks of implantation also indicate that the alkali-modified NANOZR is sufficient for use as an implant material. If biocompatibility similar to that of pure titanium metal is imparted around the NANOZR material, further modifications such as UV treatment and plasma treatment should be made in future and require thorough investigation.

## 4. Materials and Methods

### 4.1. Sample Preparation

**Figure 10:**
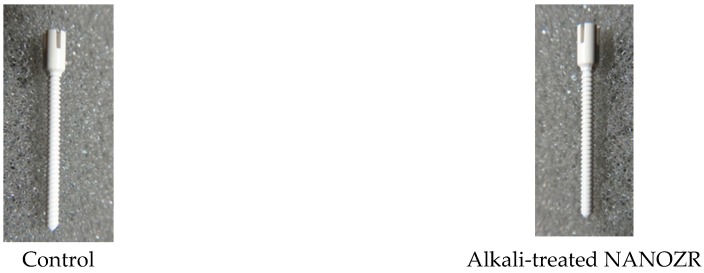
The control and alkali-treated NANOZR implant photographs.

NANOZR screw implants (1.2 mm in external diameter and 12 mm in length, Sartonworks, Kanagawa, Japan) were fabricated from NANOZR disks (Yamamoto Kinzoku, Osaka, Japan). The screw implants were immersed in 10 M aqueous NaOH at 30 °C for 24 h. The solution in each flask was replaced with 200 mL of distilled water until the solution reached a conductivity of 5 μS/cm. Samples were then dried at room temperature. Screws were divided into alkali-modified NANOZR and unmodified NANOZR groups (Figure 10).

### 4.2. Surface Characterization

SEM (S-400; Shimadzu, Kyoto, Japan) and SPM (SPM-9600; Shimadzu, Kyoto, Japan) were used to evaluate surface topography. XPS (Kratos Analytical Axis Ultra DLD electron spectrometer; Kratos Instruments, Manchester, UK) was used for evaluating the coating composition using a monochromatic Al Kα X-ray source. Argon-ion etching was performed for 2 min (evaporation rate 5 nm/min) on each sample to remove surface contaminants. ELSZ-1000ZS (Otsuka Electronics, Hirakata, Japan) were used to measure zeta potentials at 25 °C. The measurement principle is electrophoretic light scattering (laser Doppler electrophoresis), a reliable measurement based on electro-osmosis profile estimation.

### 4.3. Animal Model and Surgical Procedures

**Figure 11:**
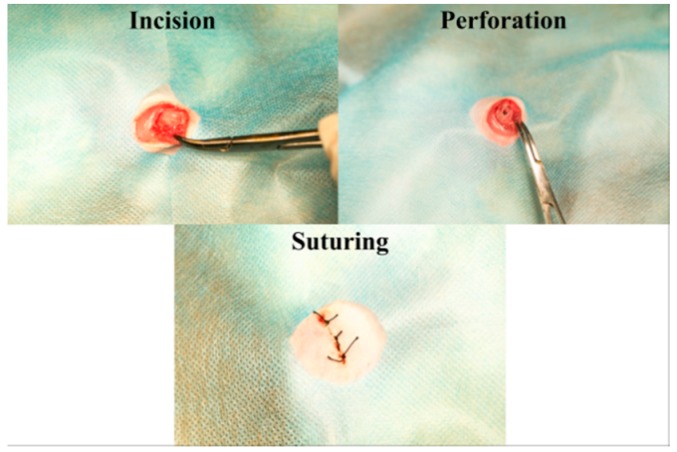
Surgical procedure.

In this study, 20 male rats (Shimizu Laboratory Supplies Co., Kyoto, Japan; age eight weeks, weighing 160 ± 15 g) were used (10 rats; alkali-modified NANOZR group, 10 rats; unmodified NANOZR group). All the animals were fed normally and in the same light cycle. Animals were anesthetized after an intraperitoneal injection of anesthetic (1.5 mL/kg). Hair was shaved from the right hind limb, the skin was disinfected first with iodine and then with 75% ethanol to remove the iodine. A longitudinal incision 1 cm in length was made along the medial side of the knee joint, and the subcutaneous fascia was incised. The patella and extensor mechanisms were then translocated to expose the distal surface of the femur. A pilot hole was drilled in the egg fossa using a 1 mm round burr burst under deep sterile saline perfusion, and the hole was expanded to 1.2 mm using an endodontic treatment file. Implants sterilized with ethylene oxide gas were randomly inserted into the 20 prepared channels and the medullary cavity of the right femur. After surgery, the knee joint was repaired and the surgical site was closed in layers. Gentamycin (1 mg/kg) and buprenorphine (0.05 mg/kg) were intramuscularly injected for three days to prevent infection after surgery and relieve pain. All rats were allowed free movement (Figure 11). This study was performed according to the Guidelines for Animal Experimentation at Osaka Dental University (Approval No. 18-03007).

### 4.4. Sequential Fluorescent Labeling

Initially, 25 mg/kg oxytetracycline hydrochloride (Sigma, St. Louis, MO, USA) was used to record the bone formation and mineralization process after implantation using bone marrow injection with fluorochrome intraperitoneal injection. Polychrome sequential labeling was performed. At Week 4, 30 mg/kg Alizarin Red S (Sigma) was injected. At Week 8, 20 mg/kg calcein (Sigma) were administered. All animals were killed by intraperitoneal injection of sodium pentobarbital three days after final labeling.

### 4.5. Alkali Treatment-Induced Bone Differentiation on NANOZR Surface In Vivo

Immediately following dissection, the right femur containing the implants was kept in cold saline and scanned with an SMX-130 CT (Micro CT) scanner (Shimadzu, Kyoto, Japan) operated at 70 kV and 118 mA. The isotropic voxel size was 10 μm in all spatial directions. After tomography, the implants and surrounding tissues were reconstructed and analyzed using morphometric software (TRI/3D-BON; Ratoc System Engineering, Tokyo, Japan). The region of interest was defined as a 500 μm wide region of bone around the implant, distal from 2 mm within the growing plate′s highest point. In the region of interest, the bone volume rate (BV/TV), the mean number of trabecular columns (Tb.N), the mean trabecular thickness (Tb.Th), and the mean trabecular spacing (Tb.Sp) were calculated.

After the micro CT scan, demineralized histological sections were prepared using femoral specimens (n = 10). The specimens were fixed in 70% ethanol solution for seven days and then immersed in Villanueva bone staining solution. Sections were analyzed by histomorphometry with a BZ-9000 digital microscope (Keyence Co., Osaka, Japan). Fluorescent microscopic evaluation was performed using a confocal laser scanning microscope (LSM 700; Carl Zeiss, Oberkochen, Germany). The excitation/emission wavelengths of oxytetracycline hydrochloride (blue), Alizarin Red S (red), and calcein (green) chelating fluorescent dye were 351/460 nm, 543/617 nm, and 488/517 nm, respectively. The measurement area was defined as approximately 2 mm below the growth plate to 1 mm distal from the growth plate based on microCT analysis. Bone area ratio (BA), bone-implant contact (BIC), and labeled bone area (LBA) were evaluated in a 200-fold field of view around the implant and sections 2 mm below the growth plate, respectively.

### 4.6. Statistical Analyses

Data are presented as the mean ± standard deviation. In all analyses, statistical significance was determined with the paired two-tailed Student’s *t*-test. A *p*-value <0.05 was considered statistically significant.

## 5. Conclusions

Our study revealed that alkali-modified NANOZR treatment is effective for hard tissue formation surrounding implants. We also showed that modification of the NANOZR implant surface by alkali treatment promoted mechanical and chemical changes. The need for in vivo experiments using larger animals such as dogs for clinical application may be considered in the near future with eventual human trials taking place.
